# Role of p53 and transcription-independent p53-induced apoptosis in shear-stimulated megakaryocytic maturation, particle generation, and platelet biogenesis

**DOI:** 10.1371/journal.pone.0203991

**Published:** 2018-09-19

**Authors:** Stephanie A. Luff, Chen-Yuan Kao, Eleftherios T. Papoutsakis

**Affiliations:** 1 Department of Biological Sciences, University of Delaware, Newark, Delaware, United States of America; 2 Delaware Biotechnology Institute, University of Delaware, Newark, Delaware, United States of America; 3 Department of Chemical and Biomolecular Engineering, University of Delaware, Newark, Delaware, United States of America; Institute of Biochemistry and Biotechnology, TAIWAN

## Abstract

Megakaryocytes (Mks) derive from hematopoietic stem and progenitor cells (HSPCs) in the bone marrow and develop into large, polyploid cells that eventually give rise to platelets. As Mks mature, they migrate from the bone marrow niche into the vasculature, where they are exposed to shear forces from blood flow, releasing Mk particles (platelet-like particles (PLPs), pro/preplatelets (PPTs), and Mk microparticles (MkMPs)) into circulation. We have previously shown that transcription factor p53 is important in Mk maturation, and that physiological levels of shear promote Mk particle generation and platelet biogenesis. Here we examine the role of p53 in the Mk shear-stress response. We show that p53 is acetylated in response to shear in both immature and mature Mks, and that decreased expression of deacetylase HDAC1, and increased expression of the acetyltransferases p300 and PCAF might be responsible for these changes. We also examined the hypothesis that p53 might be involved in the shear-induced Caspase 3 activation, phosphatidylserine (PS) externalization, and increased biogenesis of PLPs, PPTs, and MkMPs. We show that p53 is involved in all these shear-induced processes. We show that in response to shear, acetyl-p53 binds Bax, cytochrome c is released from mitochondria, and Caspase 9 is activated. We also show that shear-stimulated Caspase 9 activation and Mk particle biogenesis depend on transcription-independent p53-induced apoptosis (TIPA), but PS externalization is not. This is the first report to show that shear flow stimulates TIPA and that Caspase 9 activation and Mk-particle biogenesis are directly modulated by TIPA.

## Introduction

Megakaryocytes (Mks) are large, polyploid cells that reside in the bone marrow (BM) and differentiate from CD34^+^ hematopoietic stem and progenitor cells (HSPCs). As they mature, Mks migrate to the endothelial lining of BM sinusoids where, through gaps of the endothelium, they extend proplatelets (PPTs) into circulation [1, 2]. PPTs mature in circulation, form platelet-like particles (PLPs) leading eventually to the production of platelets, the small anuclear blood cells that regulate thrombosis, vascular repair, and immune responses [3, 4]. Understanding Mk differentiation and platelet biogenesis is important for creating safe and effective therapies for thrombocytopenic diseases, as well as for developing efficient production of platelets *ex vivo*. The demand for donor-derived platelets for transfusion continues to grow [5]. Platelet-like particles (PLPs) produced *ex vivo* would bypass the dependency on blood donors, significantly decrease the risk of contamination with blood-borne pathogens, and prevent alloimmunization to transfused platelets. Current *ex vivo* PLP production methods have low yield, which would make generating platelet doses for transfusion prohibitively expensive [6]. Understanding how Mks produce platelets *in vivo* could help to replicate the process for affordable, high-yield *ex vivo* PLP generation.

It is now firmly established that biomechanical forces are an important physiological factor in platelet biogenesis [7–9]. Notably, Mks experience complex biomechanical forces as they deform to penetrate gaps of the BM-sinusoid wall, and shear stresses upon exposure to blood flow as they extend PPTs into circulation. We have shown [9] that shear forces of physiological level accelerate DNA synthesis of immature Mks, promote phosphatidylserine (PS) externalization and caspase-3 activation, and dramatically enhance the generation of PPTs, PLPs, and Mk microparticles (MkMPs), thus promoting the overall process of thrombopoiesis. MkMPs are the most abundant microparticles (MPs) in circulation [10], and have been found to target HSPCs to induce them into Mk differentiation [9, 11]. Thus, MkMPs may potentially serve as platelet substitutes [9, 11]. Therefore, understanding the process of shear-induced thrombopoiesis and MkMP biogenesis is of both practical and fundamental importance.

In our previous report, we have shown that Mk maturation in response to shear is mediated by transcription factor AP-1 via mitogen-activated protein kinase (MAPK) mechanotransduction [12]. However, it is not yet known what regulates the cellular, apoptotic-like processes leading to Mk fragmentation in response to shear [9]. This is the focus of this present study: to examine the role of transcription factor p53 in the response of Mks to shear as it affects Mk maturation and the associated apoptotic-like processes. We discuss next why we chose to focus on p53 in this response.

Our lab has previously demonstrated that the p53 is an important regulator of megakaryopoiesis, the differentiation of HPSCs into Mks leading to mature Mks. Specifically, Furhken, *et al*. determined that p53 is responsible for controlling polyploidization and the transition to endomitosis (mitotic cycle without cytokinesis) in the megakaryocytic CHRF cell line by preventing cell cycling and stimulating apoptosis [13]. Apostolidis, *et al*. confirmed these findings *in vivo* in p53 knock out (KO) mice [14], and, in a subsequent study proposed a transcriptional regulon (including the cell cycle-regulator p21 and the apoptosis inducer Bax) to explain how p53 controls these processes [15]. The mechanism by which p53 selects the promoters of genes for either cell cycle arrest or apoptosis depends on specific post-translational modifications, and notably acetylation [16]. Acetylation at K320 drives p53 to bind to the promoters of genes for cell cycle arrest, while acetylation at K373 leads to the binding of p53 at apoptotic promoters. Acetylation at these two Lysine residues, in addition to K382, has been linked to the shear stress response of endothelial cells (ECs) [17]. K320 and K373 were deacetylated in response to shear flow in ECs, while there was increased acetylation at K382 [17]. Therefore, we hypothesized that acetylation of p53 at these sites may also occur in the Mk shear stress response.

During Mk maturation, several apoptotic regulators are engaged, altering cellular processes and structures including the plasma membrane, cytoskeleton, and nucleus [18] by activating a set of cysteine proteases known as caspases. Caspases can be activated by both intrinsic and extrinsic apoptotic pathways [19], both of which are regulated by p53 in either a transcription-dependent or -independent manner [20, 21]. Chipuk, *et al*. demonstrated that p53 induces transcription-independent p53-induced apoptosis (TIPA) by directly binding to Bax and activating it, leading to its accumulation in the mitochondria membrane and subsequent initiation of the intrinsic apoptotic pathway [21]. The main factor determining whether p53 is nuclear or cytoplasmic is its acetylation status [22]. Not only is p53 acetylation important for its nuclear export, but also for its interaction with Bax [23, 24]. Yamaguchi, *et al*. found that acetylation at K320, K373, and K382 is necessary for p53 to elicit TIPA [23]. Given that TIPA is linked to Caspase 3 activity [23] and that our lab has previously demonstrated that Caspase 3 is important in the shear-stimulated biogenesis of PPTs, PLPs, and MkMPs, we hypothesized that TIPA may be directly involved in this process. Additionally, as Caspase 9 mediates Caspase 3 activation, we hypothesized that Caspase 9 is the intermediary that links TIPA to the Mk shear stress phenotype. For the first time, we show that shear flow stimulates Bax-p53 binding, cytochrome c release, and Caspase 9 activation in Mks, and that cytoplasmic p53 plays a role in the biogenesis of Mk particles.

## Experimental procedures

### Megakaryocytic culture of CD34^+^ hematopoietic stem cells and the CHRF-288-11 megakaryoblastic cell line

Wild-type and p53-KD CHRF-288-11 (“CHRF”) cells were cultured as previously described [14, 25] for either 3 or 4 days following PMA stimulation. We confirmed that the KD CHRF cells had undetectable levels of p53 through western blot analysis (S1 Fig). Primary stem cell-derived Frozen human G-SCF mobilized peripheral blood CD34^+^ cells (Fred Hutchinson Cancer Research Center) were cultured as described [12, 26].

### Shear stress experiments

For primary stem-cell derived Mks, cells were seeded into flow slides (μ-Slide I^0.6^ Luer, Ibidi) coated with vWF (Haematologic Technologies) at 250,000 cells per slide at either day 8 or 11 [9, 12]. On day 9 or 12, cells were subjected to shear flow that resulted in shear stress levels of either 1.0 dyn/cm^2^ for 2 hours (for acetyl-p53 experiments) or 2.5 dyn/cm^2^ for 30 min (for all other experiments) using a syringe pump system established by Jiang, *et al* [9] and described in our previous study [12]. These shear levels and exposure times were examined in our previous study and are within the physiological ranges of shear Mks likely experience in the BM and lung vasculature [9]. The longer time point for probing p53 acetylation levels was chosen to ensure that entire acetylation pattern was captured, given that removal from MDM2 and phosphorylation are prerequisites to acetylation [27]. 30 min represents the time needed for trans-sinusoidal migration of murine Mk fragments into circulation [7] and is sufficient for platelet biogenesis [9]. Therefore, we performed most experiments for this duration. The respective shear flow rates for either 30 or 120 min were chosen based on previous work from our lab [9]. For all CHRF experiments, cells were seeded into the fluidic chambers one day prior to shear flow without the vWF coating (due to their increased adherence to plastic surfaces compared to primary Mks). At day 3 or 4, cells were subjected to shear flow that resulted in shear stress levels of 2.5 dyn/cm^2^ for 30 min.

### Immunofluorescence assays

Immunofluorescence staining was used for protein quantification in place of Western Blot analysis due to the low numbers of Mks retrieved after shear flow that result in sub-microgram total protein lysate, and scaling up the system was not practical. Thus, immunofluorescence coupled to MFI (mean fluorescent intensity) quantification was chosen for quantitating the levels of several proteins. Immunofluorescence staining was performed as previously described [12, 28]. Cells were fixed and stained within the flow chamber to retain morphological structures. Primary antibodies used were: anti-acetyl-p53 K320 (EMD Millipore #06–1283), anti-acetyl-p53 K373 (EMD Millipore #04–1137 or 06–916), anti-acetyl-p53 K382 (EMD Millipore #04–1146), anti-active Caspase 9 (Santa Cruz #22182), normal mouse IgG (Santa Cruz #sc-3879), normal rabbit IgG (Santa Cruz #sc-3888), and normal goat IgG (Santa Cruz #sc-2028). Secondary antibodies were anti-mouse Alexa Fluor 488 (Life Technologies #A11017), anti-rabbit Alexa Fluor 647 (Life Technologies #A21245), and anti-mouse Alexa Fluor 647 (Life Technologies #A21235). Quantification was performed within the cell perimeter only; to delineate the cell borders, 5 μM of Syto13 (Life Technologies #S7575) or Syto40 (Life Technologies #S11351) was used. Fluidic chambers were visualized using a Zeiss LSM 780 confocal microscope with Plan-Apochromat 40x/1.4 oil DIC objective and Zeiss Zen software. Immunofluorescence was quantified using Velocity software (PerkinElmer). For cells harvested from the fluidic chambers, analysis for protein quantification was performed on the FACS Aria II flow cytometer.

### Co-immunoprecipitation and western blot analysis

CHRF cells were lysed using the IP lysis buffer provided in the Dynabeads Co-IP Kit (ThermoFisher #14321D). Protein concentration was determined using the Bio-Rad DC Protein Assay Kit II (#5000112). Total p53 was immunoprecipitated with PureProteome Protein A/G Mix magnetic beads (EMD Millipore #LSKMAGAG10) and an antibody against full-length p53 (Santa Cruz #sc-6243). SDS-Polyacrylamide gel electrophoresis was performed using ExpressPlus 4–20% Bis-Tris polyacrylamide gels (Genscript #M42012) and the Mini-PROTEAN Tetra Vertical Electrophoresis Cell (Bio-Rad #1658004), followed by transfer onto nitrocellulose membrane (Genscript #L00224A60) via the Owl VEP-2 Mini Tank Electroblotting System (ThermoFisher). Membranes were blocked using 5% milk (w/v) in TBST for 1 hr at room temperature. Immunoblotting for Bax was performed using an anti-Bax primary antibody (Santa Cruz #sc-7480) and an anti-mouse Alexa Fluor 647 secondary antibody (Life Technologies #A21235) in 2.5% milk (w/v) in TBST. UV exposure of CHRF cells was performed using the UVP Blak-Ray UV lamp (#B-100SP) for 1 hr at 10J/m^2^.

In another experiment, immunoblotting for Caspase 9 with GAPDH as a reference protein was performed using anti-caspase 9 (Abcam #ab185719), and anti-GAPDH (Santa Cruz #sc-47724) primary antibodies, and anti-rabbit Alexa Fluor 647 (Life Technologies #A21245) and anti-mouse Alexa Fluor 488 (Life Technologies #11017) secondary antibodies.

### Proximity ligation assay and super resolution microscopy

The proximity ligation assay (PLA) was performed using the Duolink Red Starter Kit for Rabbit/Mouse (Sigma #DUO92101) according to the manufacturer’s specifications. Briefly, cells were fixed with 3.7% formalin (Sigma-Aldrich #F8775) for 25 min, permeabilized with 0.1% Triton X-100 (Sigma-Aldrich #X-100) for 5 min, and blocked using 0.05% Tween20 (Fisher Scientific #BP337) in 2mg/mL in PBS for 1 hour (all steps performed at room temperature). Samples were then incubated with primary antibodies against acetyl-p53 K373 (EMD Millipore #04–1137) and Bax (Santa Cruz #sc-7480), followed by incubation with the PLA probes. The Duolink PLUS and MINUS probe was used against the anti-acetyl-p53 K373 and anti-Bax antibodies, respectively. If the two proteins are interacting, the oligonucleotide PLA probes would be in proximity of each other. Ligation of the co-localized probes was performed using a ligase and amplification of the ligated oligonucleotides was achieved through rolling circle replication with a polymerase. Samples were then incubated with fluorescent probes specific for the oligonucleotides. Slides were imaged on the Zeiss Elyra PS1 Super-Resolution Microscope using structured illumination (SIM) at the Delaware Biotechnology Institute Bioimaging Center.

### Cytochrome c release assay

Cytochrome c release was measured as described in Campos, *et al*. [29]. Day 9 and Day 12 Mks were subjected to shear flow (2.5 dyn/cm^2^ for 2 hours) and then harvested from the fluidic slides using Accutase (ThermoFisher #A1110501). The plasma membrane was selectively permeabilized with Digitonin (Santa Cruz #sc-280675A). Cells were then washed in PBS to remove cytochrome c present in the cytosol (due to mitochondrial release) and fixed with 3.7% formalin in PBS. Intact cytochrome c was probed using an anti-cytochrome c antibody (Santa Cruz #sc-65396) with the immunofluorescence assay described here and was analyzed using flow cytometry (FACS Aria II, BD Biosciences).

### Transfection of primary stem cell-derived Mks

Overexpression of wild-type p53 was achieved through nucleofection using protocol T-003 on the Amaxa Nucleofector II (Lonza). “pCMV-Neo-Bam p53 wt” was a gift from Bert Vogelstein (Addgene plasmid #16434). The p53 insert was sequenced using Sanger sequencing (University of Delaware Sequencing and Genotyping Center). The S46A amino acid substitution was achieved by introducing a T136G nucleotide mutation using the Agilent QuikChange Lightning kit (#210518) with Forward (5’-TCAATATCGTCCGGGGCCAGCATCAAATCATCCAT-3’) and Reverse (5’-ATGGATGATTTGATGCTGGCCCCGGACGATATTGA-3’) mutagenic primers. Knock down of p53 in primary Mks at day 12 was achieved by transfecting CD61+ Mks at day 9 with the InvivoGen psiRNA-p53 plasmid (#psirna42-hp53) that expresses siRNA targeting p53 mRNA. Day 9 was chosen because the manufacturer suggested it takes 2 days for sufficient expression. We determined that transfection of day 11 primary stem-cell derived Mks with a control GFP plasmid achieves 70% GFP+ on day 12 (S2 Fig). Since the psiRNA-p53 plasmid does not contain a selectable marker for human cells, we performed the experiments with the assumption that a majority of Mks were successfully transfected given the 70% efficiency we can achieve. Transfected primary stem cell-derived Mks will be referred to as “p53-KD Mk”, while the previously-establish KD CHRF cells [13] will be referred to as “p53-KD CHRF”.

### Sandwich ELISAs

We performed an ELISA to confirm that p53 protein decreased following transfection of the psiRNA-p53 plasmid and that p53 protein increased following transfection of the “pCMV-Neo-Bam p53 wt” plasmid. Briefly, monoclonal mouse anti-p53 (DO-1) antibody (Santa Cruz #sc-126) was immobilized to the surface of a well in a 96-well plate (CELLTREAT #229196) in PBS buffer at room temperature (“capture antibody”). Following two washes with PBS, blocking of the remaining protein-binding sites was achieved using 5% milk (w/v) in TBST. The lysates from each sample were then incubated in each well at 37C for 90 min. Polyclonal rabbit anti-p53 (Santa Cruz #sc-6243) was then incubated in each well at room temperature for 2 hr (“probing antibody”). Following 4 washes with PBS, goat anti-rabbit Alexa Fluor 488 (Life Technologies #A11070) was incubated in each well for 1 hr. Fluorescent signal was measured using the PerkinElmer Victor 3V plate reader using an excitation/emission configuration for GFP. We also performed an ELISA to confirm that the p53-S46A mutant had decreased Bax binding. We cultured PMA-treated CHRF for 4 days and following shear-flow exposure (2.5dyn/cm^2^ for 30 min), cells were harvested and lysed in the Co-IP buffer described above. We the set up three samples to measure p53-Bax interaction: Bax capture followed by p53 probing (“Bax-p53”), p53 capture followed by Bax probing (“p53-Bax”), and p53 capture followed by p53 probing (“p53-p53”). The first two samples were designed to measure how much p53 and Bax were bound to one another while the third was to measure the total levels of p53 in the lysate. For the Bax-p53: anti-Bax antibody (Santa Cruz #sc-7480) was immobilized to the well surface, followed by incubation with the lysate, then incubation with polyclonal rabbit anti-p53 antibody (Santa Cruz #sc-6243) and goat anti-rabbit Alexa Fluor 488 secondary antibody (Life Technologies #A11070). For p53-Bax: monoclonal mouse anti-p53 antibody (Santa Cruz #sc-126) was immobilized to the well surface, followed by incubation with the lysate, then incubation with anti-Bax antibody (Santa Cruz #sc-7480) and goat anti-mouse Alexa Fluor 488 secondary antibody (Life Technologies #A11017). For p53-p53: 4% SDS (w/v) was added an aliquot of lysate to dissociate any complexes to allow for easier measurement of total p53. Monoclonal mouse anti-p53 antibody (Santa Cruz #sc-126) was immobilized to the well surface, followed by incubation with the lysate, then incubation with polyclonal rabbit anti-p53 antibody (Santa Cruz #sc-6243) and goat anti-rabbit Alexa Fluor 488 secondary antibody (Life Technologies #A11070).

In other experiments, we performed an ELISA to confirm the expression of HDAC1, p300, PCAF, and acetyl-p53. Capture antibodies include: anti-HDAC1 (Santa Cruz #sc-6299), anti-p300 (Santa Cruz #sc-32244), anti-PCAF (Sigma Aldrich #SAB1404637), and anti-p53 (Santa Cruz #sc-126). Probing antibodies include: anti-HDAC1 (Santa Cruz #sc-7872), anti-p300 (Santa Cruz #sc-584), anti-PCAF (Santa Cruz #sc-8999), anti-acetyl-p53 K320 (EMD Millipore #06–1283), anti-acetyl-p53 K373 (EMD Millipore #06–916), and anti-acetyl-p53 K382 (Cell Signaling Technology #2525). Secondary antibody used for all sandwiches was goat anti-rabbit Alexa Fluor 488 secondary antibody (Life Technologies #A11070).

### qRT-PCR assay on MDM2 expression

To examine the effect of overexpression of wild-type p53 or mutant p53 (“p53-S46A”) on the transcriptional activity of p53, 10^6^ d11 Mks were transfected with both plasmids individually with program T-003 of the Amaxa Nucleofector II (Lonza). Transfection without any additional plasmids was used as negative control (“WT”). After 24 h, total RNA was isolated from each culture with the Qiagen miRNeasy Kit following the manufacturer’s specification. After RNA extraction, 1 μg of total RNA from each group were reversed transcribed via the cDNA Reverse Transcription Kit (Applied Biosystems). qPCR assays for *MDM2* and *GAPDH* expression were performed with iTaq Universal SYBR Green Supermix (Bio-Rad) and the following primers: Forward (5’-GAGCAGGCAAATGTGCAATAC-3’), Reverse (5’-TGGTCTAACCAGGGTCTCTT-3’) for *MDM2*, and Forward (5’-CCCTTCATTGACCTCAACTACA-3’), Reverse (5’-ATGACAAGCTTCCCGTTCTC-3’) for *GAPDH*. The *MDM2* expression levels were quantified using the Livak method with normalization to the expression of *GAPDH* as a reference gene.

### Annexin V binding assay

Following shear-flow exposure, Mks were harvested from the fluidic slides using Accutase (ThermoFisher #A1110501) for 10 min at 37°C. Cells were washed twice in Annexin Binding Buffer (10 mM HEPES, 140 mM NaCl, 2.5 mM CaCl_2_, pH 7.4). Cells were then stained with anti-annexin V-PE (BD #560930) at 4°C for 15 min and analyzed by flow cytometry (FACS Aria II, BD Biosciences).

### Mk particle assays and co-culture with hematopoietic stem cells

PPTs, PLPs, and MkMPs were harvested as previously described [9, 12]. PLP quality was measured by Fibrinogen binding, as previously described [9, 12]. To assess MkMP quality, co-culture experiments were performed as previously described [9]. We chose to use CHRF-derived MPs (CMPs) because of the significant reduction of p53 (to undetectable levels) in the p53-KD CHRF strain. Following 3 days of PMA treatment, CMPs were harvested from static culture using ultracentrifugation at 25000 rpm for 30 min at 4°C and co-cultured with HSCs for 8 days. Cells were co-cultured in IMDM, 10% (v/v) BIT9500 (Stemcell #09500), 50ng/mL SCF (Peprotech), and 1X antibiotic antimycotic (ThermoFisher). At day 8, a ploidy assay was performed as previously described [13].

## Results

### Shear-forces induce increased acetylation of transcription factor p53 in immature and mature Mks

**Fig 1:**
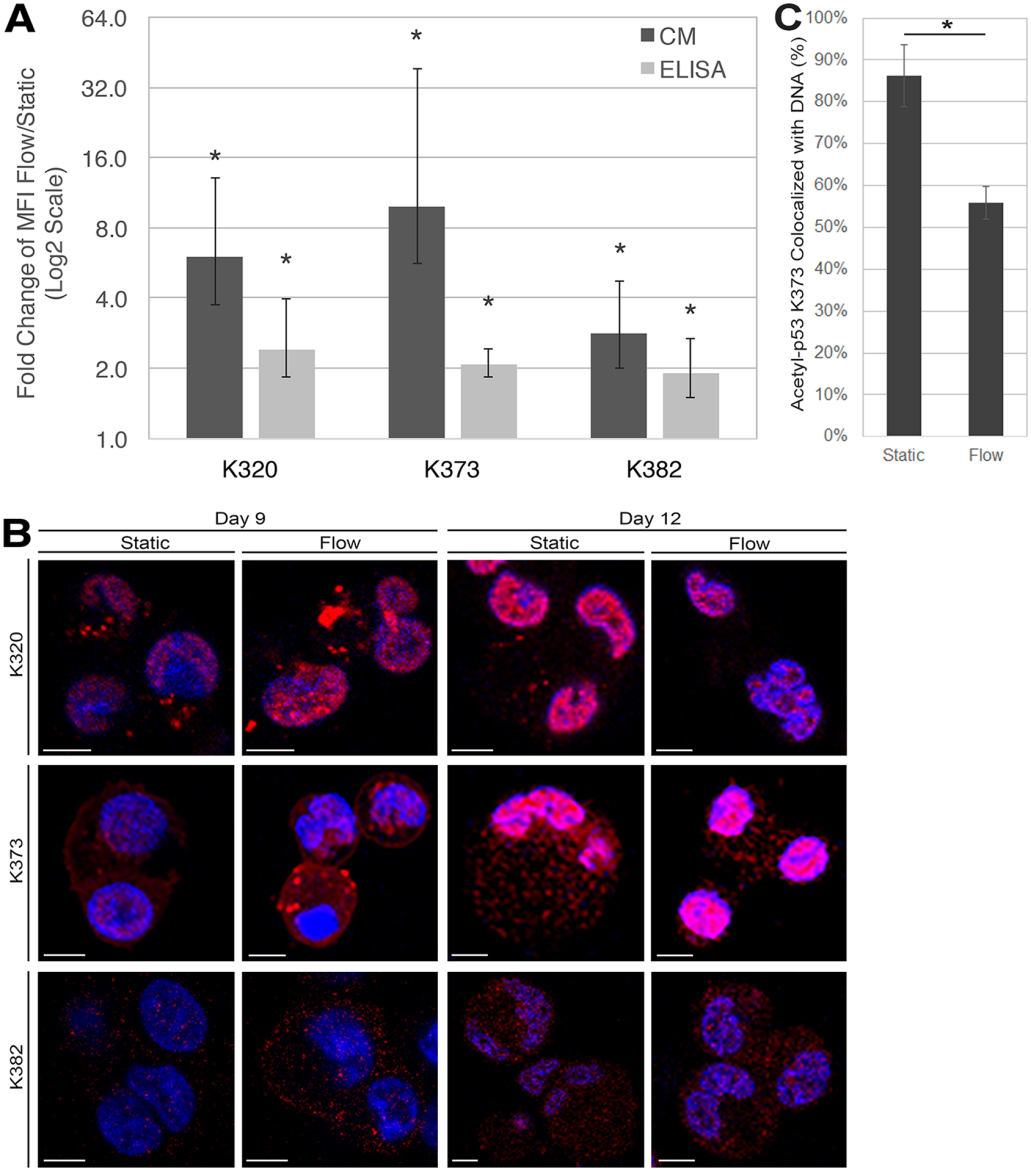
Shear flow alters p53 acetylation and sub-cellular localization in Mks. *A*, Fold change of mean fluorescence intensity (MFI) of acetyl-p53 expression for Lysine (K) 320, K373, and K382 in static and flow d9 Mks. Quantification was achieved using quantitative confocal microscopy (dark gray) and ELISA (light gray). One sample t-test, *p < 0.05, n = 3 (biological replicates) for CM, n = 4 for K373/K382 ELISA, n = 5 for K320 ELISA; error bars: SEM. *B*, Confocal microscopy of d9 and d12 Mks with immunofluorescent staining probing for acetyl-p53 (red), DAPI (blue), and co-localization (pink). C. Co-localization analysis from *B* on K372-acetyl-p53 (red) with nucleus (blue, DAPI) of d12 Mks under static or shear flow condition.

To examine whether p53 is differentially acetylated following exposure to shear flow and how it compares to what is currently known about p53 acetylation in response to shear in ECs (i.e. increased acetylation at K382 and decreased acetylation at K320 and K373) [17], we performed quantitative immunofluorescent confocal microscopy to measure the expression of acetylated p53 and determine its subcellular localization. In immature (day 9) Mks, we found significantly more acetylation of p53 at K320, K373, and K382 following shear-flow exposure by both quantitative confocal microscopy and an ELISA (Fig 1A). At both time points, Acetyl-p53 K320 was mostly localized in the nucleus (Fig 1B), under both static and shear flow conditions. A higher fraction of K373 is localized in the cytoplasm (Fig 1B), and upon shear flow, more K373 moves to the cytoplasm (less co-localization with DNA; Fig 1C). K382 was equally distributed between the nucleus and the cytoplasm (Fig 1B).

While the trend is consistent between the two quantification methods, the fold-increase detected using the ELISA (Fig 1A, light gray) is smaller than with quantitative confocal microscopy (Fig 1A, dark gray) because fluorescence intensity in an ELISA cannot be normalized to cell volume, which decreases significantly following shear flow. This is one reason quantitative confocal microscopy is necessary for accurate protein quantification of low-abundance proteins in Mks subjected to shear flow.

**Fig 2:**
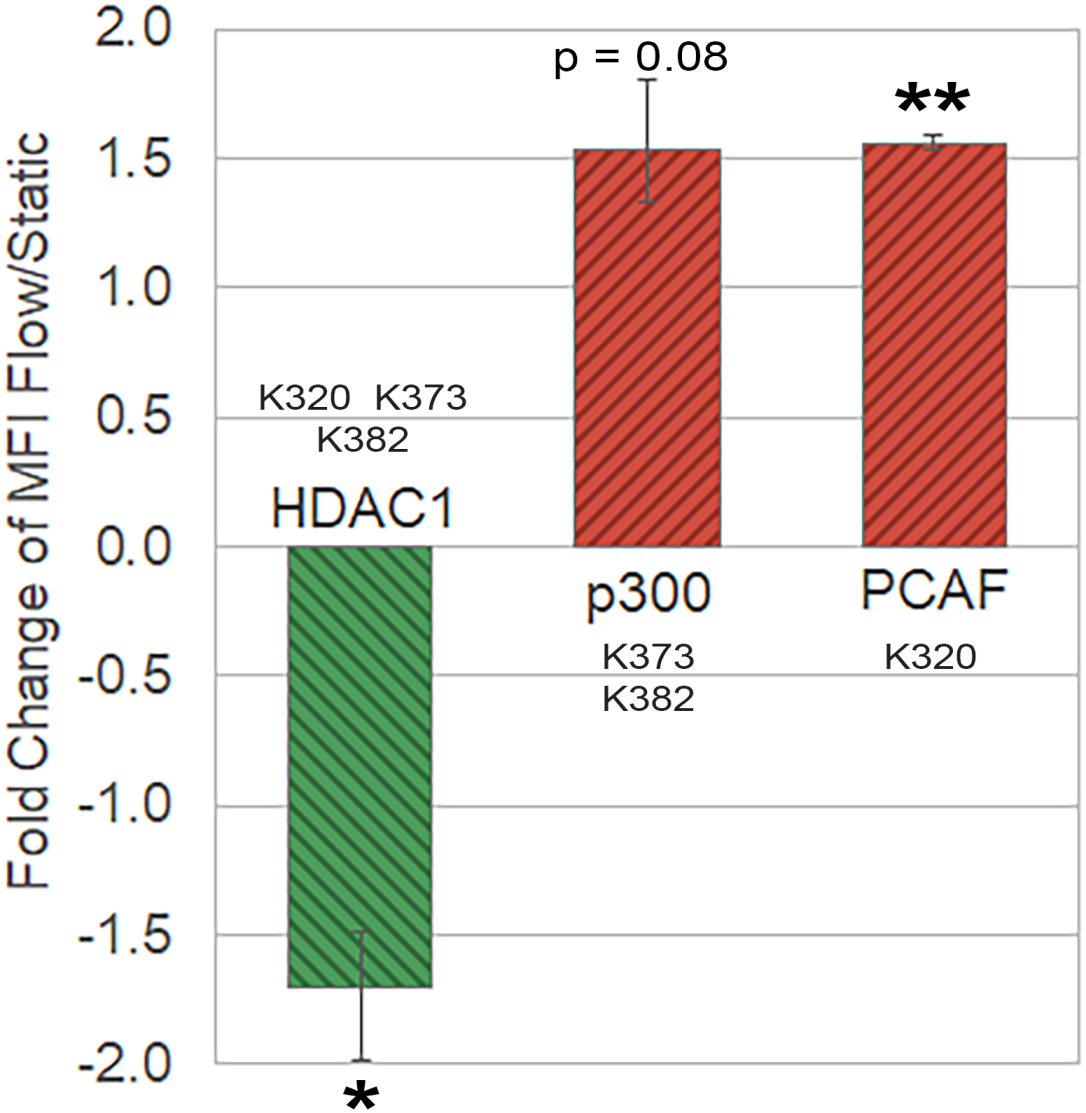
Shear flow exposure modulates expression of acetyl-modifying enzymes. Fold change of mean fluorescence intensity (MFI) in d9 Mks before and after shear-flow exposure for p53 deacetylase (HDAC1) and acetyltransferases (p300/PCAF). Quantification was achieved using an ELISA. One sample t-test, *p < 0.05, **p < 0.01. n = 3 (biological replicates), error bars: SEM. Scale bar: 5μm.

We were interested in examining the deacetylase and acetyltransferases effecting p53 to investigate potential mechanisms by which p53 acetylation is altered in response to shear flow. There is only one deacetylase, HDAC1, that removes acetyl groups from p53, but several enzymes can acetylate p53 at different residues [30]. Tip60 acetylates K120, PCAF acetylates K320, and p300 acetylates K164, K305, K370, K372, K373, K381, K382, and K386 [30]. Since K320, K373, and K382 were differentially acetylated here in response to shear, we probed the expression of PCAF and p300 acetyltransferases. As Zeng, *et al*. demonstrated that HDAC1 deacetylates p53 in the EC shear-stress response [17], we also probed the expression of HDAC1. Since differential acetylation of p53 in response to shear was substantial in day 9 Mks, we chose these cells to examine the expression of acetyl-modifying enzymes. In response to shear-flow, we found significant downregulation of HDAC1 from ELISA quantification (Fig 2), which could explain the increased acetylation at all three shear-sensitive p53 residues. In addition, there was upregulation of p300 (Fig 2), which could be responsible for the increased acetylation at K373 and K382. There was also a significant increase in PCAF expression (Fig 2), which could account for some of the K320 acetylation.

### In the shear stress response of both primary stem cell-derived Mks and megakaryocyte-differentiated CHRF cells, p53 binds and oligomerizes pro-apoptotic Bax

The cytoplasmic localization of acetyl-p53 K373 (Fig 1B) led us to explore the possibility that transcription-independent p53-induced apoptosis (TIPA) was involved in the Mk shear stress response. To pursue this hypothesis, we first examined whether the first step of this pathway, that is, p53 binding to Bax, takes place in response to shear flow. Due to cell detachment from the fluidic chamber surface during shear flow exposure, and the small number of cells in the fluidic chamber, a practically unfeasible number of primary Mk cells would be needed to perform co-immunoprecipitation (Co-IP) of p53/Bax. We resolved this difficulty by employing the Proximity Ligation Assay (PLA) in primary Mks, and Co-IP with a suitable cell line, namely the widely-validated CHRF cell line [25, 31]. PLA coupled to immunofluorescence microscopy detects endogenously-expressed protein-protein interactions on a single cell basis [32]. The PLA includes the initial steps of an immunofluorescence assay namely fixing the cells, permeabilizing the plasma membrane, and then incubating with two primary antibodies raised in different species against the two proteins of interest. Respective secondary antibodies, with conjugated oligonucleotides that are complimentary to each other, are then incubated with the samples to label the proteins of interest with each oligonucleotide. The samples are then incubated with a ligase to hybridize the oligonucleotides if they are in close proximity, followed by amplification with a polymerase by rolling circle replication. Fluorescent probes are then added to hybridize to the amplified oligonucleotides, if present. The PLA has several advantages over other techniques (e.g. FRET and double-label indirect immunofluorescence), including the ability to probe endogenously expressed proteins and increased fluorescent signal [33]. To visualize potential p53/Bax interactions at high resolution, the PLA was coupled with super resolution (structured illumination) microscopy as demonstrated by Aguullo-Pascual, *et al*. [34], and for quantification, we used quantitative confocal microscopy as described by Söderberg, *et al. [32]*.

**Fig 3:**
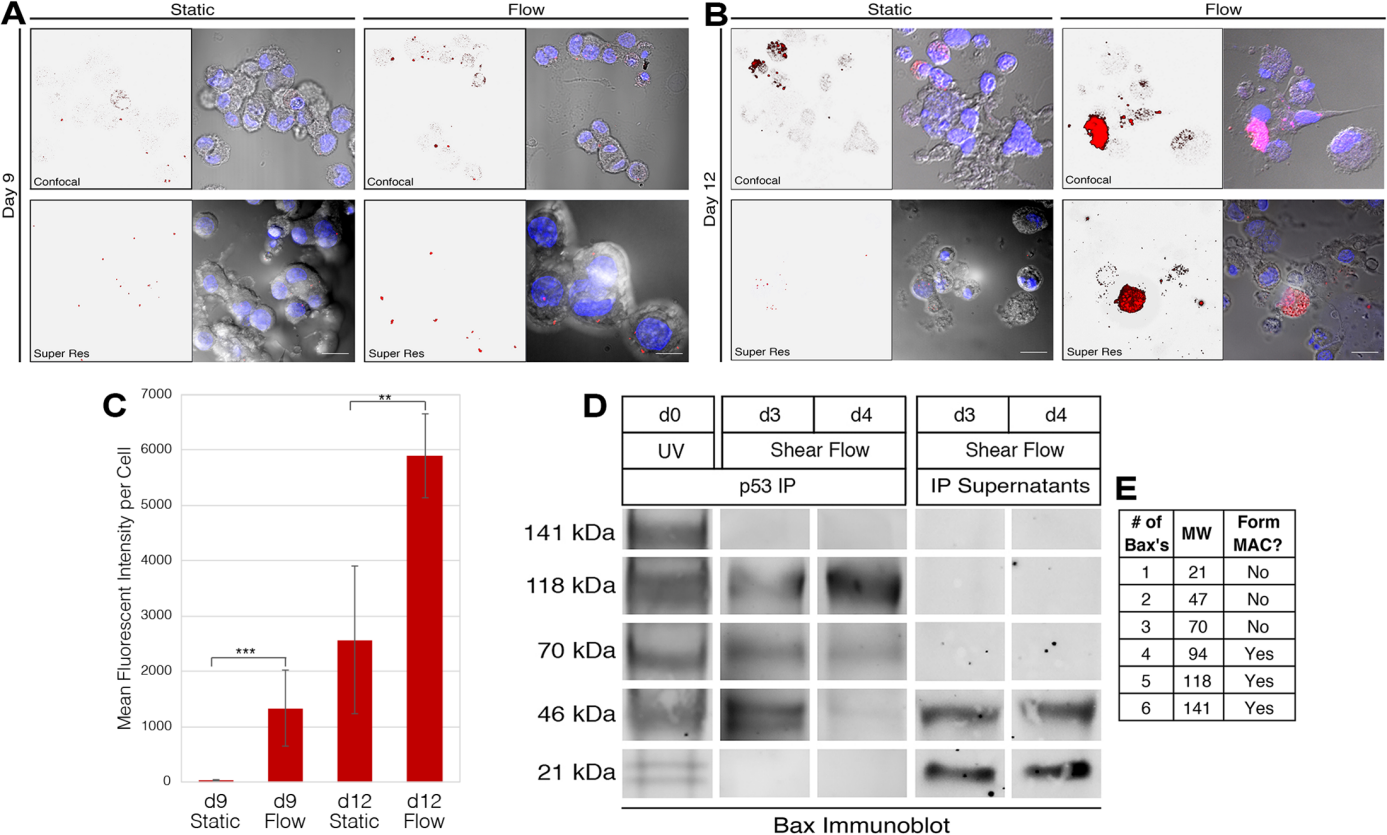
p53 binds and oligomerizes Bax in Mks following shear-flow exposure. **(***A*,*B)*, Confocal and Super Resolution microscopy (as labeled) of the Proximity Ligation Assay (PLA) between acetyl-p53 (K373) and Bax. Red: Interaction-dependent fluorophore; Blue: DAPI. Scale bar: 5μm. *C*, MFI of interaction-dependent fluorophores from the confocal microscopy images shown in *A and B*. The cell number for quantification vary from 10 to 20. Statistical analysis is performed by Student’s t-test, **p < 0.01, ***p < 0.001. *D*, Co-immunoprecipitation of p53 immunoprecipitation and Bax immunoblot demonstrating the interaction between the two proteins, as well as the oligomeric status of Bax in the complexes. *E*, Hypothesized molecular weights (MW) for each oligomer of Bax, as determined by Antonsson, *et al*. [35], and whether the respective Bax oligomer can form a mitochondrial apoptosis-induced channel (MAC), as determined by Saito, *et al*. [36].

In this PLA assay, interaction between Bax and p53 resulted in fluorescent punctations, which are clearly visible on the super resolution microscope. As shown in Fig 3A and 3B, there were some p53-Bax interactions in statically-grown Mks, suggesting there is a basal level of interactions in unstressed cells. Following shear-flow exposure, the number of interactions increased in both day 9 and day 12 Mks, demonstrating that shear flow stimulates interactions above the basal level (Fig 3A). Punctations were larger at day 12, suggesting that multiple interactions may be near each other (e.g. at the mitochondria) or that the Bax oligomers are larger (Fig 3B). We also see very bright signal in some day 12 Mks that were not observed in day 9 Mks (Fig 3B). Through quantitative confocal microscopy, we were able to quantitate the intensity of the PLA reactions in each image and found that shear flow significantly increased the PLA signal at both time points (Fig 3C). Interestingly, PLA signals in both static and flow samples were higher at day 12 than their respective day 9 samples (Fig 3C). This suggests that p53/Bax interactions increase with Mk maturation irrespective of the exposure to shear, thus implicating such interactions in Mk maturation.

We supported the PLA findings with a Co-IP in the megakaryoblastic CHRF cell line. Collecting a large number of CHRF cells following shear exposure was not a limiting factor as we can generate a large number of these cells and furthermore, because these cells are more adherent than primary Mk cells, we can collect many more cells after shear exposure for the IP assays. When the *in vitro* cell line was developed by Fugman, *et al*., it was determined that CHRF cells express Mk markers but are not biphenotypic for erythroid markers [37], indicating that they are past the megakaryocytic-erythroid progenitor (MEP) time point. In the primary stem cell-derived Mk culture protocol used here as developed by Panuganti, *et al*., late progenitors (such as the MEP) arise around days 5–7 of culture [26]. Using transcriptomic data from both CHRF and primary Mk cells as reported by Fuhrken, *et al*. [25], we determined that days 3 and day 4 of PMA-treated CHRF cells correspond to days 9 and day 12, respectively, of primary stem cell-derived Mks. Our Co-IP (p53 immunoprecipitation and Bax immunoblot) data in CHRF cells differentiated into megakaryocytes show that shear-flow exposure stimulated Bax binding to p53 at both days 3 and 4 (Fig 3D). At day 3 (which is analogous to day 9 Mks), the most prevalent oligomer immunoprecipitated was the dimer, while at day 4 (analogous to day 12), it was the pentamer (Fig 3D). The estimated molecular weights of Bax oligomers, determined by Antonsson, *et al*. using SDS-PAGE [35], are summarized in Fig 3E. We also performed an immunoblot for Bax in the IP supernatants (the protein not pulled down by p53 immunoprecipitation) and found that the unbound Bax were monomers or dimers (Fig 3D). We hypothesized that shear flow would activate only a subset of apoptotic processes, but not necessarily induce the full cascade of events leading to cell death. To determine how shear flow compares to a strong inducer of apoptosis, we also performed a Co-IP in CHRF cells exposed to UV irradiation. Following UV exposure, most of the p53-bound Bax proteins were high molecular weight oligomers, including the 141kDa hexamer not seen following shear-flow exposure (Fig 3D). Co-immunoprecipitation of Bax with p53 was significantly lower in static Mks (S3 Fig). While the PLA detected low levels of interaction due to its increased sensitivity compared to Co-IP, these results together suggest interaction in static Mks is possible but infrequent compared to Mks subjected to shear flow. These data support the idea that the apoptotic events stimulated by shear flow are different from the canonical apoptosis events induced by genotoxic damage.

### Shear flow induces partial release of cytochrome c from the mitochondria of mature Mks

Once Bax is inserted into the outer mitochondrial membrane, it oligomerizes to form a channel [38]. Apoptotic stimuli also induce cytochrome c release from the mitochondrial inner membrane, releasing it in the intermembrane space of the mitochondria [39]. The channel formed by Bax oligomers, called the mitochondrial apoptosis-induced channel (MAC), allows for cytochrome c to be released from the intermembrane space into the cytoplasm [38, 39]. Since the Bax oligomers following shear flow were different from the oligomers observed after UV exposure (Fig 3D), measuring the amount of cytochrome c released from the mitochondria would inform whether shear flow activates apoptotic processes through the intrinsic apoptosis pathway. Using a flow cytometric assay developed by Campos, *et al*. [29], we measured the amount of cytochrome c released in response to shear flow. The method involves selectively permeabilizing the cytoplasmic membrane (while leaving the mitochondrial membranes intact), washing the cells to remove cytochrome c present in the cytoplasm, and then performing an immunofluorescence assay to measure the amount of remaining cytochrome c, which would be expected to be intact in the inner mitochondrial membranes.

**Fig 4:**
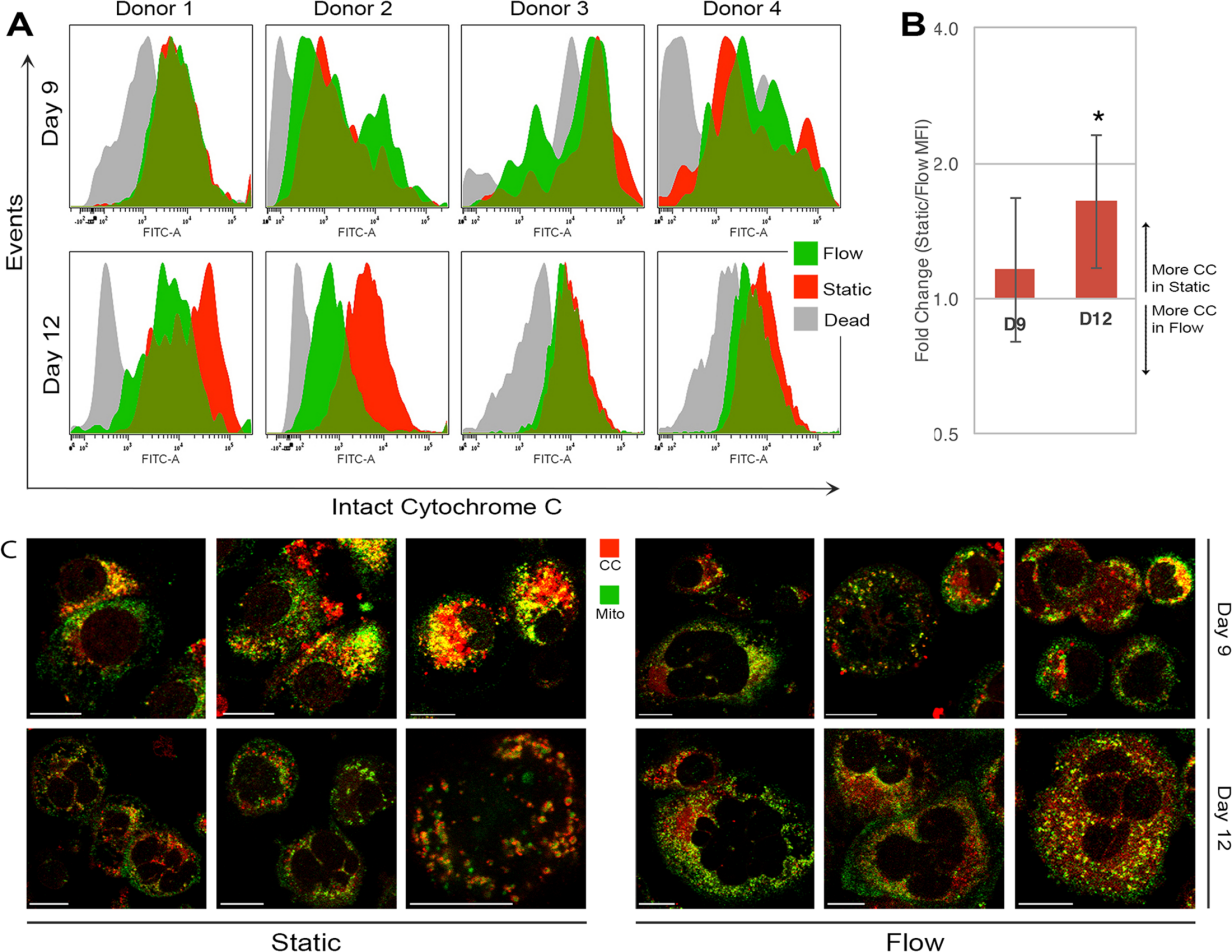
Mitochondria from immature and mature Mks subjected to shear flow have different levels of intact cytochrome c. *A*, Histograms showing cytochrome c expression in live static Mks (red), live Mks following shear flow (green), and dead Mks (gray). A peak shift to the left demonstrates decreased cytochrome c in the mitochondria membrane. *B*, Average fold change (static/flow) of mean fluorescence intensity of the histograms shown in *A* for day 9 (left) and day 12 (right). *p < 0.05, one-sample t-test, n = 4 (biological replicates), error bars: SD. *C*, Immunofluorescence confocal microscopy of d9 (top) and d12 (bottom) Mks probing for cytochrome c (red) and mitochondria (green); colocalization of the two appear as yellow (yellow). Scale bar: 10μm.

As shown in Fig 4A and 4B, our data suggest that most of the cytochrome c remains intact at day 9 before and after shear-flow exposure. This is supported by confocal microscopy analysis that demonstrates a strong overlap of cytochrome c and mitochondria signal (shown as yellow in Fig 4C). At day 12, shear flow stimulated a measurable release of cytochrome c, indicated by a peak shift to the left and statistically significant decrease in MFI (Fig 4A and 4B). Consistent with the peak shift, there was less yellow signal in the confocal microscopy images (Fig 4C). Interestingly, the confocal microscopy data show that the statically-grown Mks at day 12 had less intact cytochrome c than the statically-grown Mks at day 9 (Fig 4C). We cannot compare the MFI values between day 9 and day 12 Mks because the latter are larger and there is no method for normalizing values to cell size. The static and flow histograms in Fig 4A were compiled using only live cells. To demonstrate the validity of the assay, we also show the histograms for dead cells (which have lower forward- and side-scatter on the flow cytometer): as expected, dead cells have a substantial release of cytochrome c (Fig 4A). Taken together, these data suggest that shear flow stimulates cytochrome c release in day 12 Mks.

### Shear flow stimulates activation of Caspase 9 in both immature and mature Mks, which occurs concurrently with PS externalization

**Fig 5:**
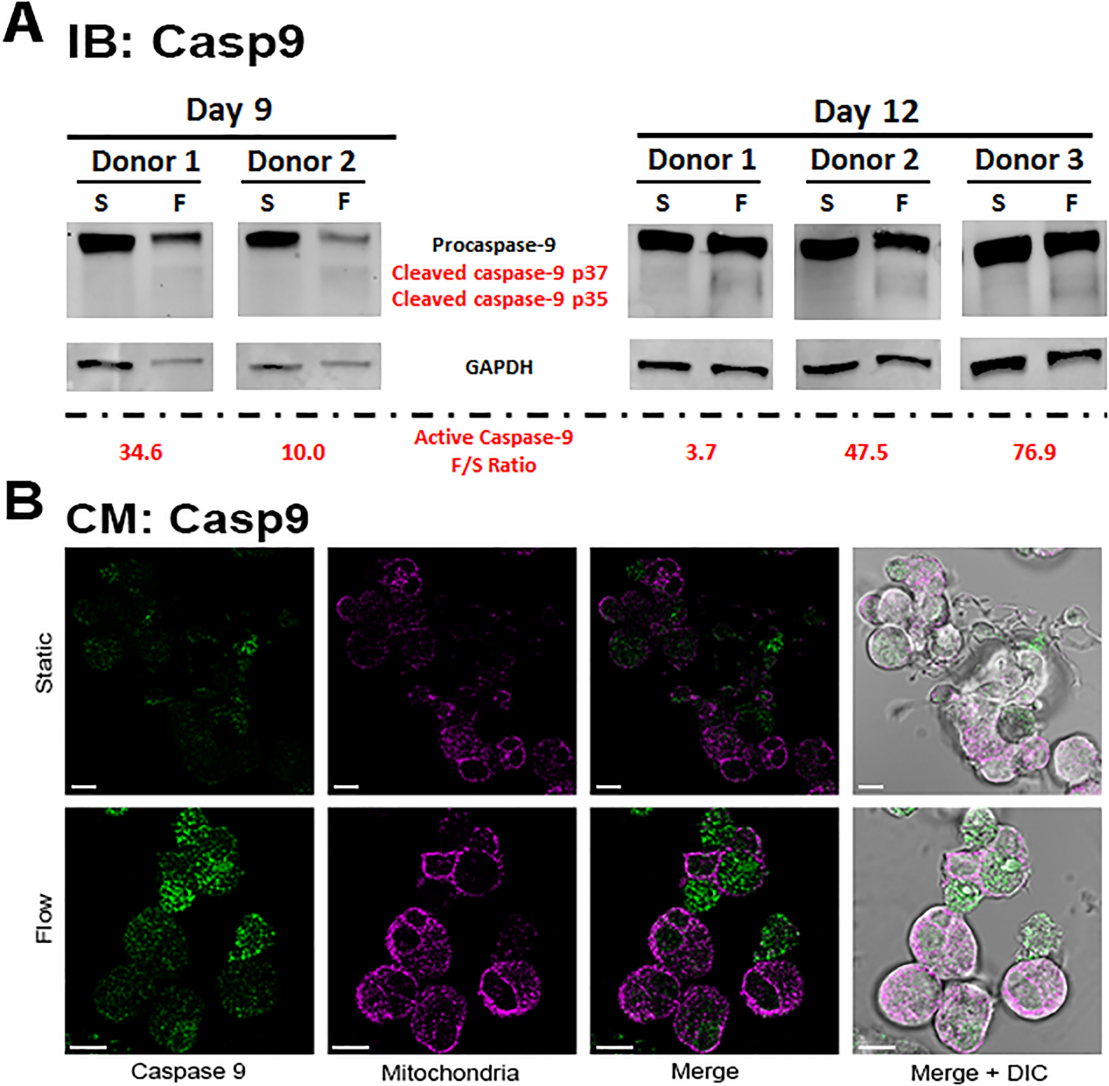
Caspase 9 activation in Mks is enhanced upon shear-flow exposure. *A*, Expression of active (cleaved) Caspase 9 expression from d9 and d12 Mks was quantified via immunoblot (Western-blot) analysis. Quantification was performed by Image J with normalization to expression of GAPDH, a reference protein. *B*, Confocal microscopy of representative d12 Mks before (top row) and after (bottom row) shear-flow exposure. Green: active Caspase 9, Pink: MitoTracker. Scale bar: 10μm.

Our lab has previously shown that shear flow stimulates Caspase 3 activation in Mks and that as the percentage of PPT-producing Mks increased, so did the expression of active Caspase 3 [9]. Additionally, pan-Caspase and Caspase 3 inhibition during Mk maturation significantly decreases PLP/PPT production [9]. In order to determine if transcription-independent p53-induced apoptosis (TIPA) is involved in shear-induced Caspase 3 activation, we examined the activation of Caspase 9, which is upstream of Caspase 3 activation and downstream of cytochrome c release [19]. To do this, we examined the expression of active Caspase 9 in immature (day 9) and mature (day 12) Mks using Western-blot analysis (Fig 5A, S4 Fig) and confocal microscopy (Fig 5B) and found that following shear-flow exposure, active Caspase 9 (cleaved Caspase 9) expression increased at both days.

**Fig 6:**
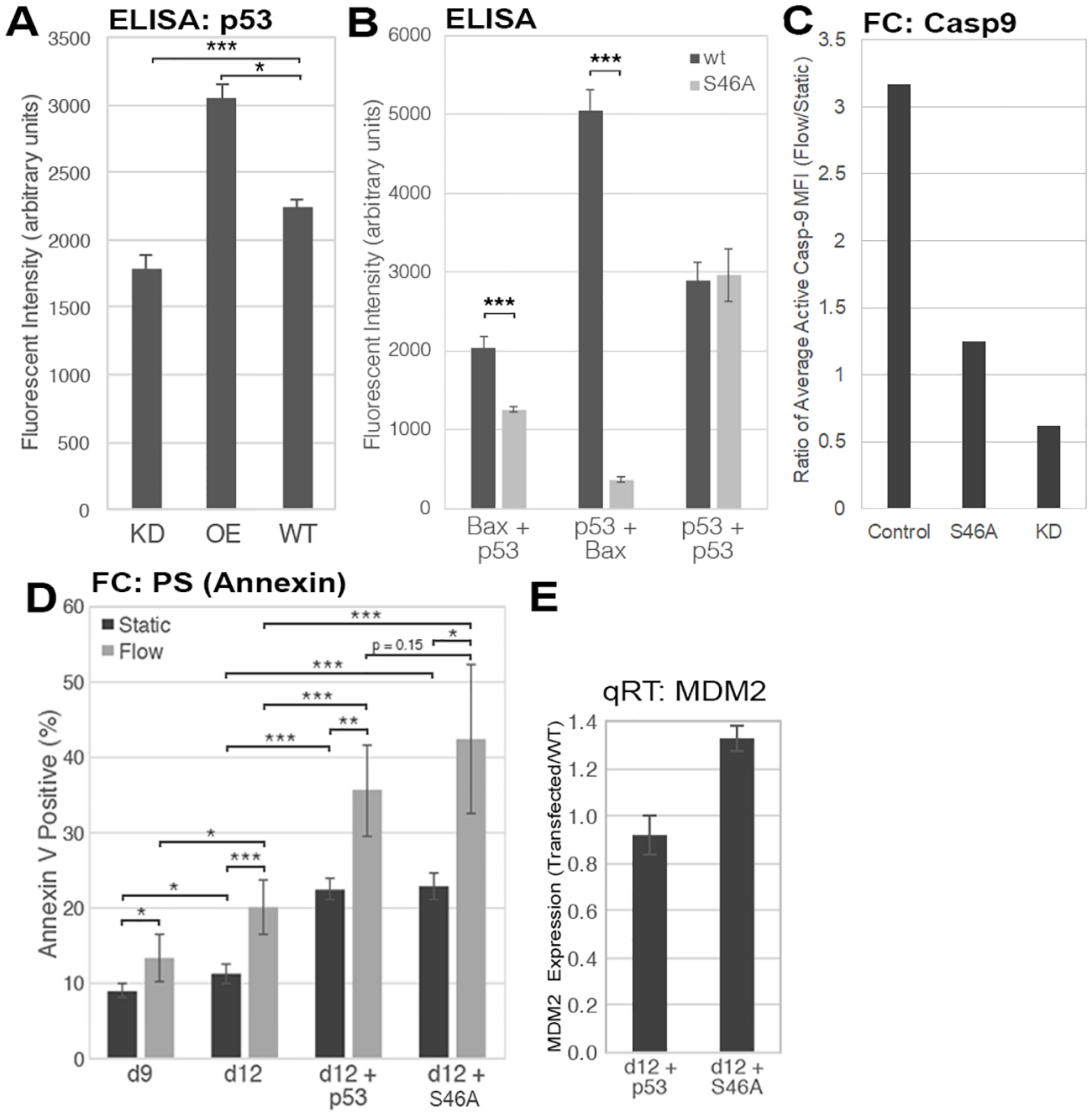
Shear-induced Caspase 9 activation in Mks is TIPA-dependent while Shear-induced PS externalization is p53-dependent, TIPA-independent. *A*, ELISA measuring total p53 levels in p53-KD, p53-overexpression, and wild-type Mks and normalized to basal lysate fluorescence. Unpaired *t*-test, n = 3 (technical replicates), *p < 0.05, ***p < 0.001, error bars: SD. *B*, ELISA measuring the amount of p53 bound to Bax (left), Bax bound to p53 (center), and total p53 in the sample (right) in wild-type CHRF cells (black) and CHRF cells expression p53-S46A (gray) following shear flow exposure at d4 post-PMA treatment. Fluorescence values were normalized to basal lysate fluorescence. Unpaired *t*-test, n = 3 (technical replicates), ***p < 0.001, error bars: SD. *C*, Percent of wild-type, p53-overexpression, p53-S46A-expressing, or p53-KD Mks at d12 with detectable expression of active Caspase 9 before (black) or after (gray) shear-flow exposure as measured by flow cytometry. Unpaired *t*-test, n = 3 (biological replicates), *p < 0.05, **p < 0.01, error bars: SD. *D*, Percent of cells expressing PS on their plasma membrane (Annexin^+^) in wild-type d9 and d12 Mks, Mks with wild-type p53 overexpression, and Mks expressing mutant p53-S46A before (black) or after (gray) shear-flow exposure. Unpaired *t*-test, n = 3 (biological replicates), *p < 0.05, **p < 0.01, ***p < 0.001, error bars: SD. *E*, Expression levels of *MDM2* following overexpression of wt-p53 or expression of mutant p53-S46A quantified by qRT-PCR.

To examine the impact of p53 on Caspase 9 activation, we knocked down (KD) and overexpressed wild-type (WT) p53, and also expressed a mutant form of p53 (see below) in primary Mk cells. As probed by an ELISA assay, p53 KD reduced the level of p53 protein by 21% while p53 overexpression resulted in 36% increase in p53 protein (Fig 6A). p53 overexpression was optimized to increase the levels of p53 protein without extensive cell death (S5 Fig). To determine the role of p53/Bax interaction on Caspase 9 activation, we designed a mutant form of p53 (“p53-S46A”) that does not bind Bax as detailed below. The mutation was achieved using site-directed mutagenesis using the same method others have used for mutating cytoplasmic p53 [23, 24, 40, 41]. The mutant p53 was created by a Serine-to-Alanine substitution using site-directed mutagenesis against Serine 46 of the wild-type p53 plasmid used for overexpression [42]. We chose S46 based on the structural modeling work by Follis, *et al*. that demonstrated the importance of this residue in Bax binding [43]. We performed an ELISA to validate the functions of p53-S46A and confirmed that the mutation severely impaired the ability of Bax to bind p53 (Fig 6B).

We used these different Mk populations (WT, p53-overexpression, and p53-S46A-expressing) to determine whether TIPA is involved in Caspase 9 activation. We assume that the expression of p53-S46A will generate a discernible phenotype even in the presence of the WT p53. We wanted to understand how the three cell populations differed, so we used the same immunofluorescence protocol for staining active Caspase 9 but measured on the flow cytometer instead. We chose to examine day 12 Mks because our goal was to understand TIPA in the context of Mk-particle biogenesis, which is not found at day 9. Expression of p53-S46A and p53-KD decreased, in a statistically significant way, Caspase 9 activation compared to WT and p53-overexpression Mks (Fig 6C), although the difference between p53-S46A and control was not as large possibly because of the presence of the WT p53 as discussed above. These findings further implicate p53 in Caspase 9 activation.

We also wanted to elucidate whether PS externalization is modulated by TIPA and if it occurs concurrently with Caspase 9 activation. Consistent with previous findings by Jiang, *et al*. [9], we confirmed that shear flow exposure increased PS externalization in immature and mature WT Mks (Fig 6D). Overexpression of WT p53 significantly increased PS expression in both statically-grown Mks and Mks exposed to shear flow at day 12. Also, the phenotype in which shear flow stimulates PS externalization was conserved upon p53 overexpression (Fig 6D). This is consistent with previous findings from our lab that found p53 modulates PS externalization in CHRF cells and primary murine Mks [13, 44]. While these data demonstrate that PS externalization is p53-dependent, we also wanted to examine the role of TIPA in this process. To do this, we used the p53-S46A mutant protein that does not bind Bax. Given the effects of p53-S46A on Caspase 9 activation and that overexpressing p53 increased PS externalization, we hypothesized that expressing p53-S46A would negate PS externalization. However, we unexpectedly found that expressing p53-S46A did not decreased PS externalization in static Mks or Mks subjected to shear flow as comparted to the p53-overexpressing Mks (Fig 6D). In fact, expression of p53-S46A enhanced PS externalization under both static and shear conditions similar to that of the p53 overexpression thus suggesting that PS externalization is affected by p53 but not by TIPA.

In order to confirm that these effects were caused by non-transcriptional events, we examined the mRNA levels of *MDM2*, which is regulated by p53. Others have shown that the p53-S46A mutation can increase the expression of *MDM2* [45]. We observed a slight increase (30%) in *MDM2* expression in the Mks with p53-S46A compared to Mks overexpressed with WT p53 (Fig 6E). In contrast, Mayo, *et*. *al* found significantly greater expression of *MDM2* (estimated 3-fold increase), with a negligible change in MDM2 protein expression and a limited cellular response (20% increase in cellular survival) following transfection with p53-S46A [45]. Given how small is the change in *MDM2* expression here in comparison, it likely has no or very little impact on the responses we see in the Mks with p54-S36A.

For the first time, these data suggest that TIPA is necessary for shear-induced Caspase 9 activation and that PS externalization occurs concurrently with TIPA but is not directly regulated by it. The only other study to examine Caspase 9 activation in the context of TIPA showed no direct involvement of p53, but instead an indirect link, and examined these processes in cells not relevant to primary Mks, namely the HCT116 human colorectal carcinoma cell line [46]. Our data presented here lead us to conclude that shear flow stimulates Caspase 9 activation in a TIPA-dependent manner but that shear-induced PS externalization is TIPA-independent.

### Transcription-independent p53-induced apoptosis is important in the shear-stimulated Mk-particle generation and the biological effectiveness of these particles

**Fig 7:**
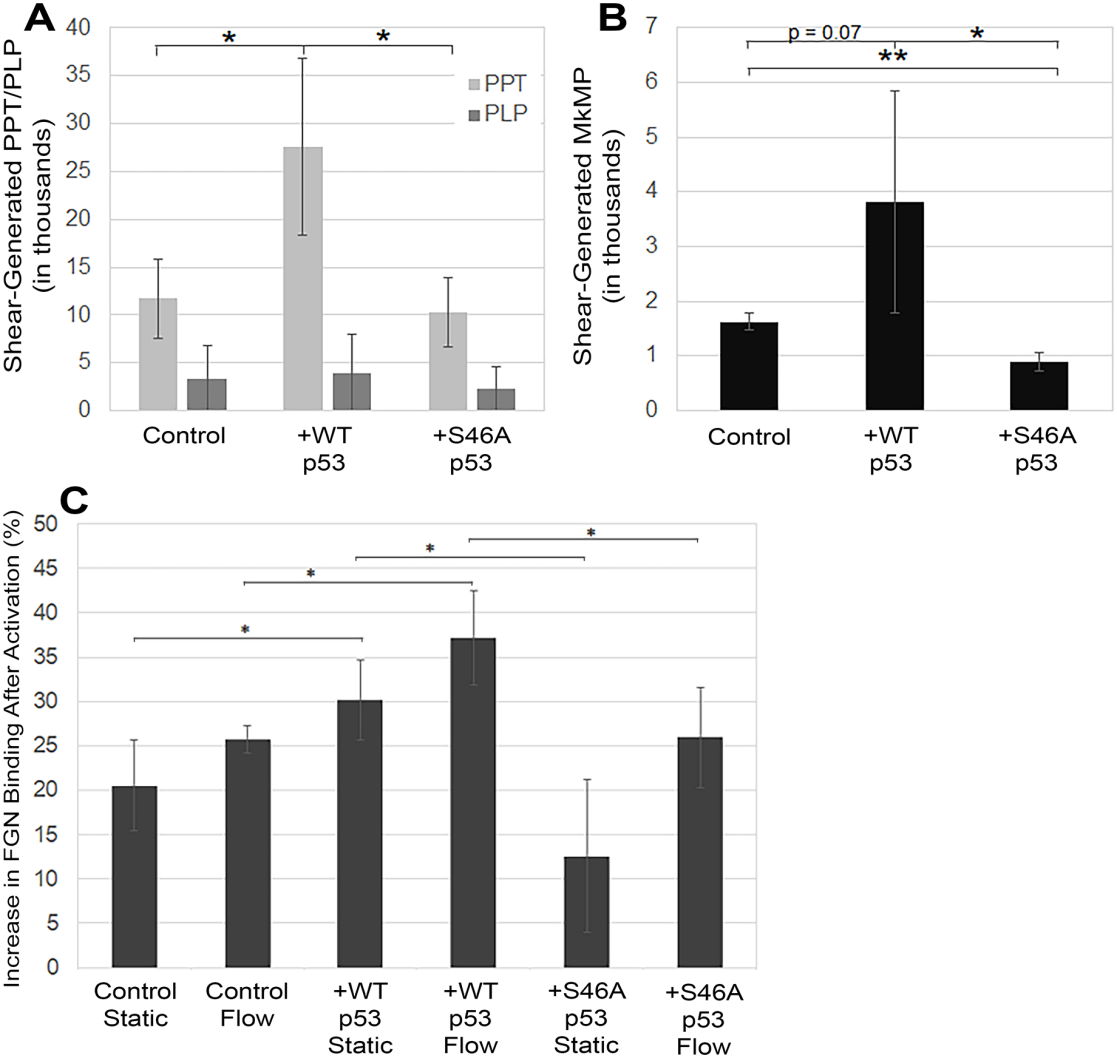
Shear-stimulated Mk particle biogenesis is dependent upon transcription-independent p53-induced apoptosis. *A*, Number of PPTs and PLPs generated by shear-flow exposure from wild-type, p53-overexpression, and p53-S46A Mks. Particle numbers were normalized per 100,000 Mks. Unpaired t-test, *p < 0.05, n = 3 (biological replicates), error bars: SD. *B*, Number of MkMPs generated following shear-flow exposure by wild-type, p53-overexpression, and p53-S46A Mks. MkMP numbers were normalized per 100,000 Mks. Unpaired *t*-test, *p < 0.05, ***p < 0.001, n = 3 (biological replicates), error bars: SD. *C*, Percent of PLPs from wild-type (WT), p53-overexpression (OE), and p53-S46A (d46) Mks that are bound to fibrinogen (FGN) following activation with 3 units of thrombin. Unpaired t-test, *p < 0.05, n = 3 (biological replicates), error bars: SD.

To determine the importance of shear-stimulated TIPA on Mk maturation, we examined how modulating its activity affected the biogenesis of Mk particles (PPTs, PLPs, and MkMPs) from day 12 Mks. Our lab has previously shown that shear flow stimulates Mk particle generation in a caspase-dependent manner [9]. Given the data we present here that links cytoplasmic p53-bax interaction with Caspase 9 activation, we hypothesized that TIPA may have a direct role in the biogenesis of these particles. To test this hypothesis, we overexpressed WT p53 and expressed the p53-S46A mutant to examine whether shear-stimulated particle biogenesis was impacted. Overexpression of wild-type p53 increased the number of PPTs and MkMPs generated by shear-flow exposure (Fig 7A and 7B), indicating that the level of p53 expression may play a role in Mk particle generation under shear-flow. Expression of p53-S46A decreased the number of PPTs generated by shear flow to the level of WT Mks (Fig 7A), while for MkMPs it decreased the number significantly lower than those generated by WT Mks. Since the mutant p53 was unable to bind to Bax, this result confirmed the importance of the interaction between p53 and Bax (Fig 3), indicating that TIPA was involved in mediating Mk particle biogenesis. While the number of PLPs generated by mature Mks was not affected by the p53 modulation (Fig 7A), their ability to bind fibrinogen (FGN) increased in p53-overexpression Mks and decreased to that of wild-type in Mks expressing p53-S46A (Fig 7C). FGN binding is an important functional feature of platelets and PLPs and indicates the likely effectiveness of platelets and PLPs in an *in vivo* setting. To examine the role of p53 acetylation in shear-induced Mk particle biogenesis, inhibition of p53 acetyltransferases resulted in decreased shear-induced PLP generation by 19% and 27% with 1 μM and 10 μM of acetyltransferase inhibitor, respectively. From these data, we conclude that TIPA is directly involved in the production of these Mk particles mediated by p53 acetylation. Taken together with the data of Fig 3 showing that p53 acetylation enhanced p53-bax interaction, and the data in Fig 7, that p53-bax interactions play a role in shear-stimulated Mk particle biogenesis, these results demonstrate the importance of p53 acetylation in TIPA-induced Mk particles biogenesis after shear flow exposure.

**Fig 8:**
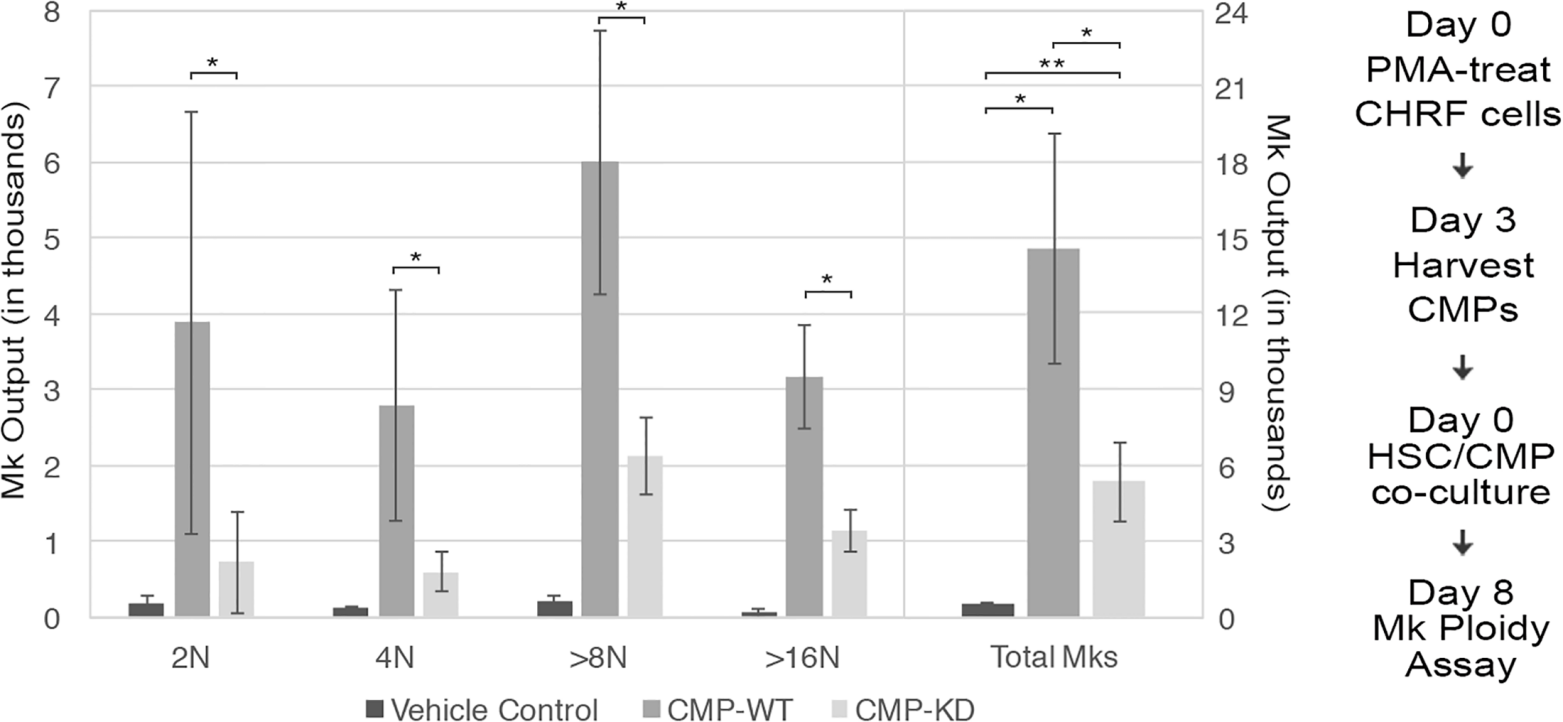
CHRF-derived MPs (CMPs) from p53-KD CHRF cells induce megakaryocytic differentiation of HSCs to a lesser extent than CMPs from wild-type CHRF cells. Number of Mks in each ploidy class and overall (as labeled) following and 8-day co-culture of CD34^+^ HSCs with vehicle control (black) or CMPs from wild-type (dark gray) or p53-KD (light gray) CHRF cells; Unpaired *t*-test, *p > 0.05, **p > 0.01, n = 4 (biological replicates).

Previous studies from our lab have demonstrated the target specificity of MkMPs for HSPCs and their biological impact on inducing Mk differentiation of HSPCs [9, 11]. Since we determined that TIPA is involved in MkMP biogenesis, we hypothesized that TIPA might also play a role in the quality of MkMPs in terms of their ability to program HSPCs. To examine this, we co-cultured CD34^+^ HSPCs with CHRF-derived MPs (CMPs) from WT and p53-KD CHRF cells [13]. As shown below, CMPs can also induce Mk differentiation of HSPCs, although less strongly so compared to MkMPs. We chose to use CMPs in this part of the work for simplicity and cost effectiveness as we can quickly and inexpensively generate CMPs in contrast to MkMPs. As expected, co-culture with WT CMPs induced Mk differentiation of CD34^+^ HSPCs; however, CMPs from p53-KD CHRF cells displayed attenuated function (Fig 8). The number of CD41^+^ Mks induced by co-culture with CMPs from p53-KD CHRF cells decreased significantly (Fig 8). All ploidy classes had significantly decreased numbers of Mks, demonstrating that expansion of Mks in all ploidy classes was negatively impacted (Fig 8). This confirms that TIPA plays a role not only in the number of MkMPs produced (Fig 7) but also the “quality” of these MkMPs in their ability to program HSPCs toward megakaryocytic differentiation in the absence of thrombopoietin (Tpo).

## Discussion

For this study, we hypothesized that p53 is involved in the physiologically significant shear-stress response of Mks during Mk maturation and thrombopoiesis for three reasons: p53 is a well-characterized modulator of apoptosis [47], is important for Mk maturation [13–15, 44], and plays a role in the shear stress response of ECs [17]. There are several studies demonstrating that cytoplasmic p53 binds and activates Bax, leading to its translocation and insertion into the mitochondrial membrane, thus initiating the intrinsic apoptosis cascade [21, 48].

Here, we have shown that p53 is differentially acetylated in response to shear, but the acetylation pattern is different from that of ECs. In response to shear flow in ECs, the p53 Lysine residues K320 and K373 were deacetylated, but there was increased acetylation at K382 [17]. In this study, we found (Fig 1A) that all three Lysine residues displayed increased acetylation in response to shear in immature day 9 Mk cells. These differences make sense given the profoundly different phenotypic responses to shear of ECs and Mks. In ECs, this specific acetylation pattern is important for inducing a quiescent state in response to laminar flow [17], while in Mks, it is responsible for creating the complex phenotype as described here and our prior work [9, 12]. To some extent, p53 acetylation occurs independent of shear flow between days 9 and 12, as we see increased acetylation in mature Mks at day 12 (Fig 1B). Given the low abundance of acetyl-p53 at day 9 compared to day 12 (Fig 1B), it is logical that shear flow would stimulate acetylation of p53 only in day 9 Mks (Fig 1A). While it is known that hyperacetylation induces cytoplasmic localization of p53 [22] and that preventing acetylation blocks Bax binding [23], it is not currently known whether p53 acetylation serves a specific role, other than increasing proximity, for inducing Bax interaction. Shear flow may modulate this interaction either by increasing p53 acetylation in Mks in earlier days (represented by the data of Day 9) or by translocating more acetyl-p53 K373 to the cytoplasm (where the interaction with Bax takes place) at later days represented by the data of Day 12.

To elucidate whether p53 binding to Bax in Mks following shear induces downstream apoptotic events, we probed the amount of mitochondrial-bound cytochrome c present in day 9 and 12 Mks (Fig 4C). Our data suggest only a partial release of cytochrome c following shear at day 12, which has been previously documented [49, 50]. Ott, *et al*. determined that the amount of cytochrome c released from the mitochondria is dictated by its interactions with cardiolipin, the phospholipid responsible for anchoring cytochrome c to the inner membrane [51]. The partial release of cytochrome c at day 12 may be modulated by this interaction and the incomplete oligomerization of Bax, leading to a controlled released aimed at stimulating Mk maturation, rather than inducing cell death.

We also examined how transcription-independent p53-induced apoptosis (TIPA) is involved in the Mk shear stress response of immature (day 9) and mature (day 12) Mks. While several studies have implicated intrinsic apoptosis in the maturation of statically-grown Mks [52–55], there have been limited studies that examine the role it plays in more physiologically-relevant culture systems, such as following shear-flow exposure. Previous work in our lab had shown that enhanced Mk-particle biogenesis, Caspase 3 activation, and PS externalization, which can result from activation of the intrinsic apoptosis cascade [19], are observed in Mks following shear-flow exposure [9]. However, it had not yet been determined how these events were being initiated in response to shear flow. To examine the role of TIPA, we modulated TIPA activity by expressing the p53-S46A mutant that cannot bind Bax. We found that TIPA was involved in the number of PPT and MkMPs and the “quality” of PLPs and MkMPs. Interestingly, as shown in Fig 7B, expression of p53-S46A decreased the number of MkMPs below that of wild-type (this trend was also seen with Caspase 9 activation; Fig 6C). Although the Mks still expressed wild-type p53 from their genomes, it is possible that the p53-S46A had a dominant negative function against the wild-type p53. Mayo, *et al*. discovered that a Serine-to-Alanine mutation at S46 (the same mutation performed to prevent Bax binding) targeted nuclear p53 to the promoter of *MDM2* (the gene that encodes the p53 inhibitor) instead of promoters for apoptotic genes [45]. This would increase the number of WT p53 molecules in inhibitory complexes with MDM2 and possibly explain how expression of the mutant p53 would decrease Caspase 9 activation and MkMP biogenesis below that of WT Mks.

Our data have shown that shear-induced Caspase 9 activation and Mk-particle generation are TIPA-dependent, while PS externalization is likely TIPA-independent. It is possible that shear-induced PS externalization occurs independent of any apoptotic mechanism. Several studies have demonstrated that PS externalization can occur without the engagement of apoptotic cascades [56, 57]. Our previous study showed that the JNK and p38 signaling pathways were important for transducing shear-flow stimuli in Mks [12] and inhibition of JNK causes delayed PS externalization [58]. Therefore, it is possible that shear-induced PS externalization is directly modulated by the JNK pathway.

JNK may also be involved in activating p53 prior to acetylation in Mks. Phosphorylation stabilizes p53 by preventing its MDM2-modulated inhibition and can be a necessary prerequisite to acetylation [27]. The JNK kinase has been shown to phosphorylate p53 at the Threonine 81 residue. Interestingly, Pluquet, *et al*. have shown that agents inducing p53 phosphorylation by JNK resulted in a cytoprotective phenotype rather than leading to cell death [59]. This is consistent with our findings that suggest that despite the stimulation of specific apoptotic effectors to modulate Mk-particle biogenesis, immediate cell death following shear-flow exposure appears to be avoided (evidenced by the differing Bax oligomer status compared to UV-exposed cells and differential cytochrome c staining compared to dead cells; Figs 3D and 4A). The data we present here suggest that shear flow stimulates a less potent form of apoptosis as compared to strong apoptotic damage. Clarke, *et al*. discovered that while the mitochondria in the cell body of mature, proplatelet-forming Mks were no longer viable, mitochondria near and within proplatelets were still metabolically active [60]. This dichotomy of active and inactive mitochondria may explain how a dying Mk cell body can produce viable pre-platelets and platelets. Our results presented here provide some insight into how this contrasting phenotype is achieved. For the first time, we demonstrated that TIPA is possible in primary human myeloid cells and that TIPA is directly involved in Caspase 9 activation. Since these findings are the first to directly link p53 in transcription-independent Caspase 9 activation, additional studies will confirm whether the findings are reproducible and consistent across multiple cell types and cell stressors, and whether additional factors are involved.

**Fig 9:**
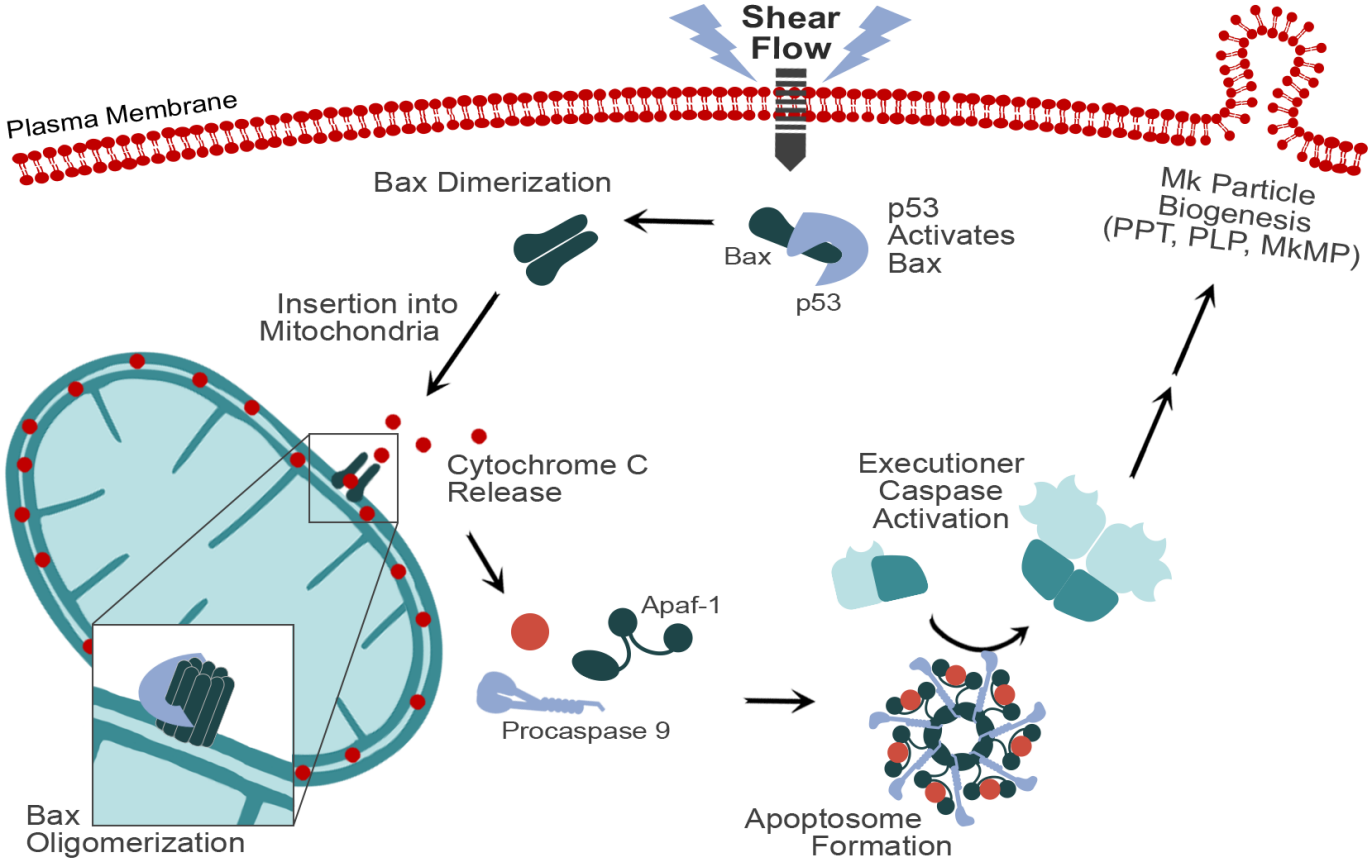
Intrinsic apoptosis cascade involving Bax dimerization, cytochrome c release, apoptosome formation, and Caspase activation. Shear flow stimulated transcription-independent p53-induced apoptosis (TIPA) and initiated the intrinsic apoptosis cascade by binding Bax, which is known to induce Bax dimerization in the cytosol and cause translocation to the mitochondria [61]. Bax then oligomerizes to form a channel, allowing for the release of cytochrome c, which was observed following shear-flow exposure. Cytochrome c, Procaspase 9, and Apaf-1 bind to form the apoptosome complex [62], followed by auto-cleavage of Procaspase 9 into Caspase 9. Our data indicate that shear flow stimulates the cleavage of Procaspase 9. The apoptosome with active Caspase 9 can cleave and activate executioner Caspases (i.e. Caspase 3). Previous work in the lab demonstrated that shear-stimulated Caspase 3 activation was important for the biogenesis of Mk particles (PPT/PLP/MkMP) [9]. Our data here directly implicated TIPA in these processes.

The information provided by this study could be useful for optimizing *in vitro* culture of primary stem-cell derived Mks for the purpose of generating high yields of high-quality PLPs and MkMPs. This information may also explain physiological and pathophysiological phenotypes that relate to the role of p53 and shear flow in normal or aberrant thrombopoiesis and MkMP biogenesis, and inspire strategies for physiological or pharmacological interventions. A physiological phenotype, such as the role of physical activity in enhanced thrombopoiesis, is discussed in [9]. Several pathophysiological phenotypes, including phenotypes related to the large number of p53 mutations in a variety of cancer phenotypes, have been discussed by us previously [13, 44]. As depicted in a visual summary in Fig 9, our data demonstrate that shear flow stimulates the binding of p53 to Bax, release of cytochrome c from the mitochondria, and Caspase 9 activation. We also demonstrated that TIPA is involved in shear-induced Caspase 9 activation and the biogenesis of MkMPs. We also discovered that while shear-induced PS externalization is p53-dependent, it is not likely TIPA-dependent. These findings provide context to previous observations from our lab that discovered shear flow induced Caspase 3 activation and MkMP biogenesis.

## Supporting information

S1 Fig Western blotting analysis of p53 expression in CHRF cells with p53 knock-down (p53-KD) or p53-KD followed by rescuing with wild-type p53. p53 level was presented as percentage of control (WT cells).
(TIF)

S2 Fig Transfection efficiency from pmaxGFP-transfected or negative control d12 Mks. GFP expression was analyzed by flow cytometry.
(TIF)

S3 Fig Uncropped western blot for Fig 3. (TIF)

S4 Fig Caspase 9 activation in Mks is enhanced upon shear-flow exposure. Expression of active (cleaved) caspase 9 and inactive Procaspase 9 expression from d9 and d12 Mks was quantified via immunoblot (Western-blot) analysis. GAPDH was used a reference protein.
(TIF)

S5 Fig Representative images of wild-type, p53 knock-down, or p53 overexpression d12 Mks. (TIF)

## Abbreviations

BM: bone marrow
EC: endothelial cell
HSPC: hematopoietic stem and progenitor cell
KD: Knock Down
KO: Knock Out
Mk: Megakaryocyte
MkMP: Mk-derived microparticle
MP: microparticle
PLP: Platelet-Like Particle
PPT: Pro/Pre-Platelet
PS: phosphatidylserine
TIPA: transcription-independent p53-induced apoptosis
Tpo: thrombopoietin
