# Supplementary figures and images for: Role of p53 and transcription-independent p53-induced apoptosis in shear-stimulated megakaryocytic maturation, particle generation, and platelet biogenesis
Source: PLoS One. 2018 Sep 19;13(9):e0203991. doi: 10.1371/journal.pone.0203991 (PMC6145578; doi:10.1371/journal.pone.0203991)

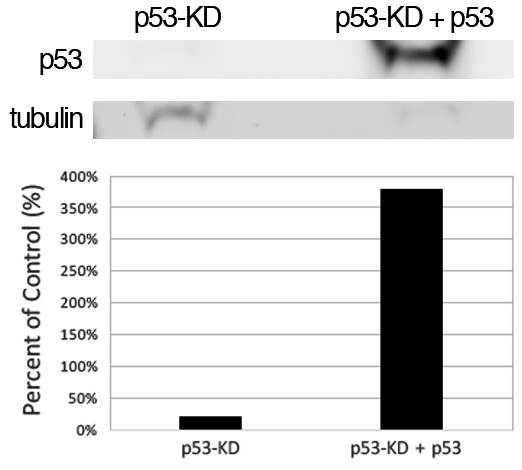

Supplement: S1 Fig — p53 level was presented as percentage of control (WT cells). (TIF) [file pone.0203991.s001.tif]

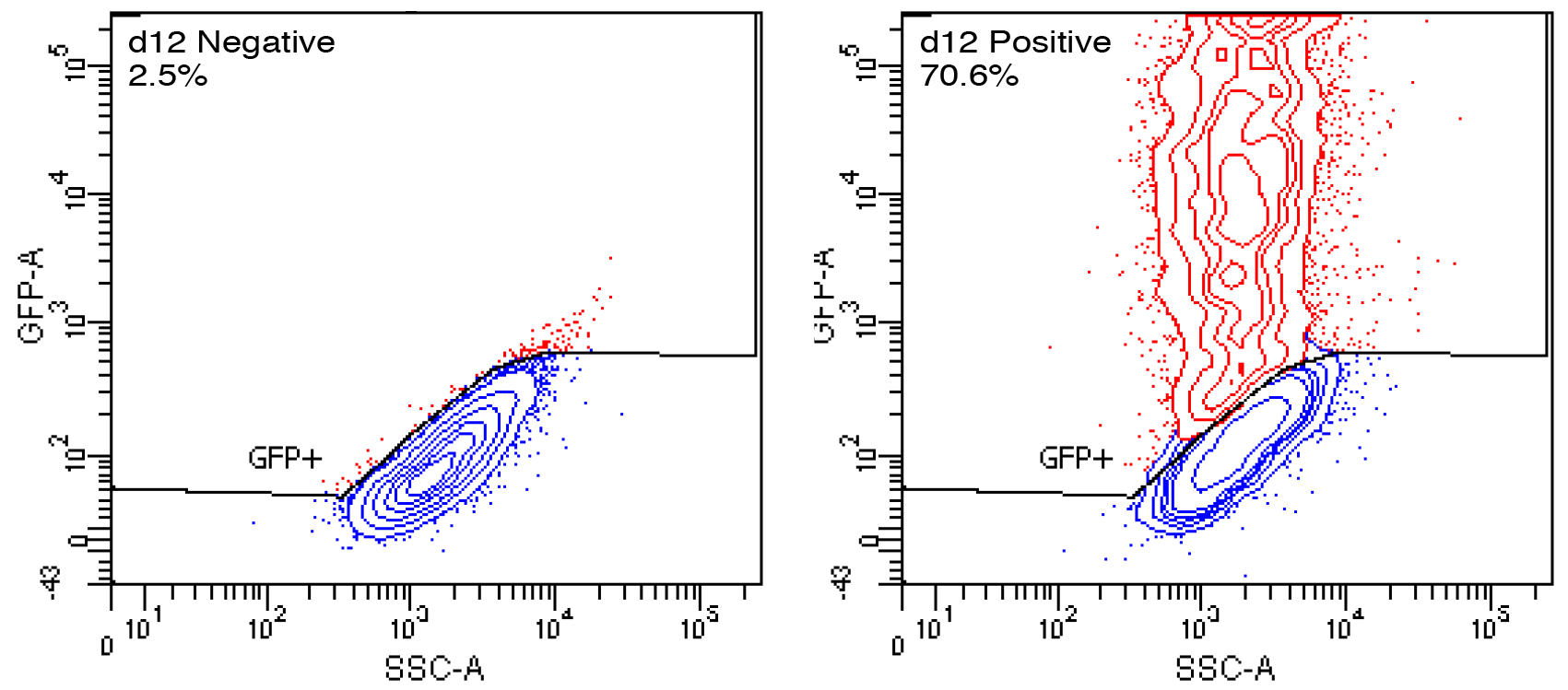

Supplement: S2 Fig — GFP expression was analyzed by flow cytometry. (TIF) [file pone.0203991.s002.tif]

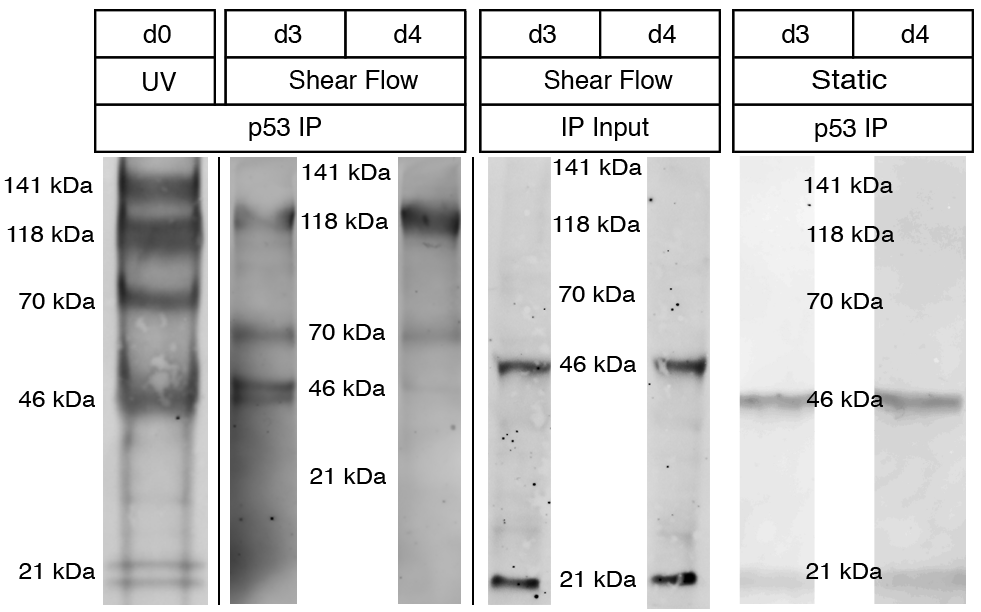

Supplement: S3 Fig — (TIF) [file pone.0203991.s003.tif]

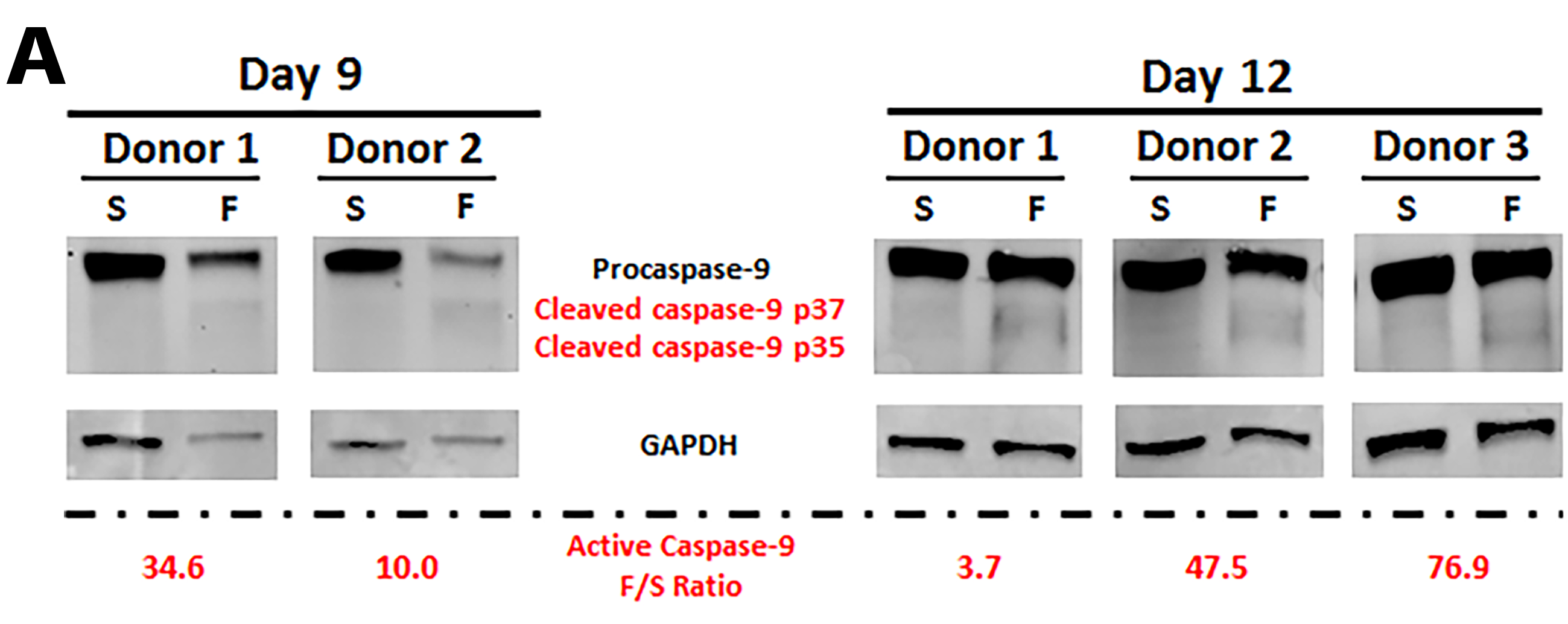

Supplement: S4 Fig — Expression of active (cleaved) caspase 9 and inactive Procaspase 9 expression from d9 and d12 Mks was quantified via immunoblot (Western-blot) analysis. GAPDH was used a reference protein. (TIF) [file pone.0203991.s004.tif]

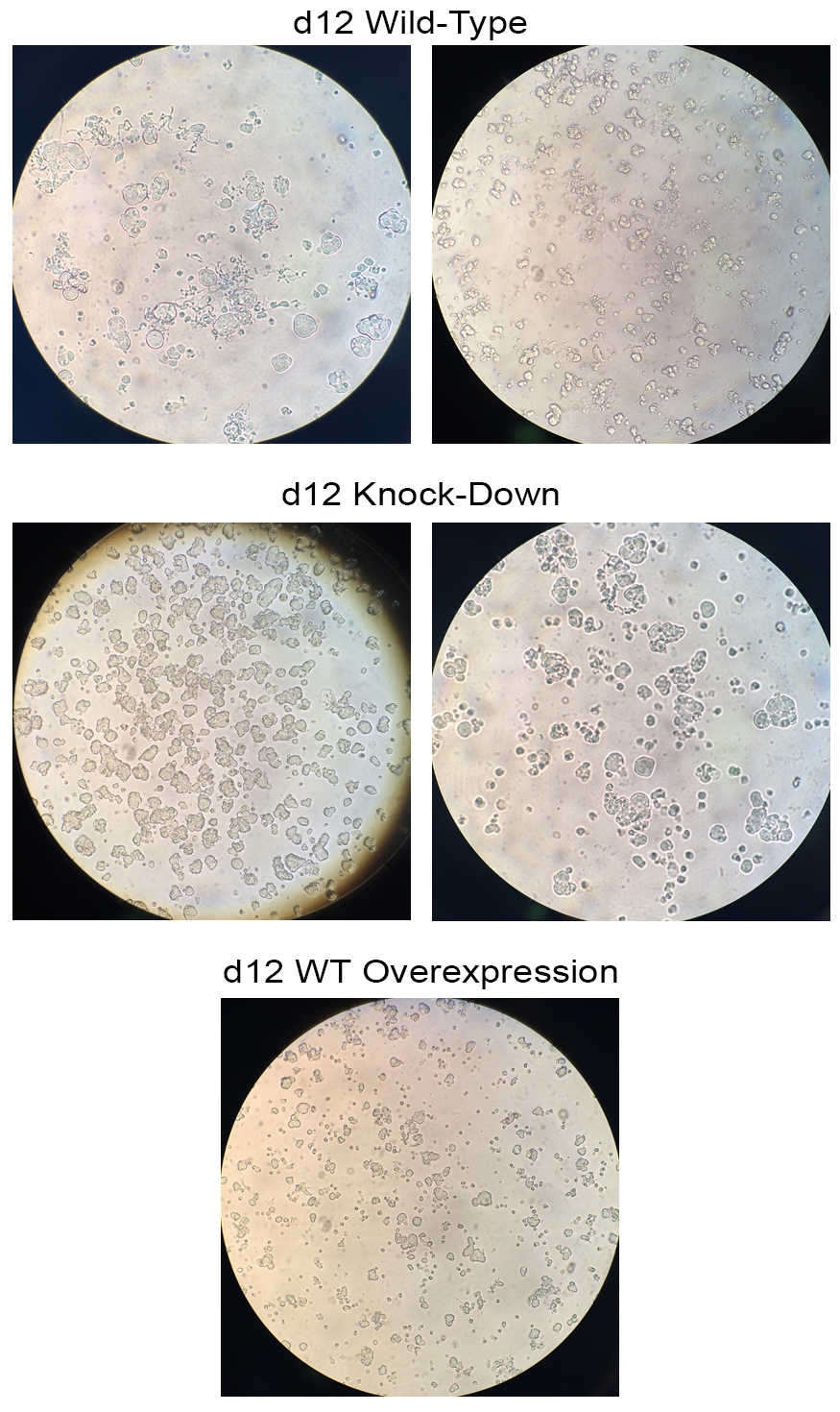

Supplement: S5 Fig — (TIF) [file pone.0203991.s005.tif]
